# Nitroxoline resistance is associated with significant fitness loss and diminishes *in vivo* virulence of *Escherichia coli*

**DOI:** 10.1128/spectrum.03079-23

**Published:** 2023-12-08

**Authors:** Felix Deschner, Timo Risch, Claas Baier, Dirk Schlüter, Jennifer Herrmann, Rolf Müller

**Affiliations:** 1 Microbial Natural Products, Helmholtz Institute for Pharmaceutical Research Saarland (HIPS), Helmholtz Centre for Infection Research (HZI) and Department of Pharmacy Saarland University, Saarbrücken, Germany; 2 German Centre for Infection Research (DZIF), Braunschweig, Germany; 3 Institute for Medical Microbiology and Hospital Epidemiology, Hannover Medical School (MHH), Hannover, Germany; Nevada State Public Health Laboratory, Reno, Nevada, USA

**Keywords:** nitroxoline, antibiotic resistance, fitness, virulence, proteomics, metabolism, genome analysis, urinary tract infection

## Abstract

Nitroxoline (NTX) is an antibiotic approved for the treatment of uncomplicated urinary tract infections (UTIs) caused by Enterobacteriaceae like *Escherichia coli*, and it has been on the market for more than 50 years. Despite being in use longer than several other clinically relevant antibiotics, the resistance of clinical isolates against NTX has not evolved significantly. To better understand this observation, we performed a standardized *in vitro* evaluation of NTX in comparison to other UTI drugs with a focus on resistance development and especially their consequences. We have managed to generate low-level resistant mutants in *E. coli* and *Klebsiella pneumoniae* using a long-term exposure setup, and identified mutations in different efflux-related genes and in other pleiotropic regulators as well as the sensor histidine kinase *envZ*. Subsequently, mutants were characterized by metabolic and proteomic analyses confirming major effects of NTX resistance on both metabolism and differential protein expression of *E. coli*, which ultimately also translates into reduced *in vitro* motility and *in vivo* virulence of mutant strains as shown in a zebrafish larvae infection model.

**IMPORTANCE:**

Antimicrobial resistance (AMR) poses a global threat and requires the exploration of underestimated treatment options. Nitroxoline, an effective broad-spectrum antibiotic, does not suffer from high resistance rates in the clinics but surprisingly, it is not heavily used yet. Our findings provide compelling evidence that Nitroxoline resistance renders bacteria unable to cause an infection *in vivo*, thereby reinvigorating the potential of Nitroxoline in combating AMR.

## INTRODUCTION

Antimicrobial resistance (AMR) and the emergence of new multidrug-resistant (MDR) pathogens is a major threat to public health (1, 2). First-line treatment options become ineffective while reserve antibiotics rise in importance resulting in almost 5 million deaths globally associated with AMR in 2019 (3, 4). Hence, the World Health Organization repeatedly calls for development of new antibiotics preferably addressing new targets. Nitroxoline (5-nitro-8-hydroxyquinoline, NTX), however, is a rather old antibiotic and has been on the European market for more than half a century. It was approved for the treatment of uncomplicated urinary tract infections (uUTIs) caused by *Escherichia coli* or *Klebsiella pneumoniae*. Other recommended treatment options are fosfomycin (FOS), nitrofurantoin (NFT), pivmecillinam (PIV), or trimethoprim (TMP), whereas the latter is only recommended with reservations due to increasing resistance rates (5). Despite being in use longer than several other clinically relevant antibiotics, Nitroxoline resistance (NTX^R^) does not seem to be a major clinical problem, and it is still recommended as first-line treatment option (5 – 9).

Chelation of biologically relevant metal cations is believed to be a central aspect of the NTX mechanism of action, as it was shown to bind to zinc, cadmium, manganese, and copper (10, 11). Cation-binding potentially results in inhibition of the RNA polymerase by cofactor deprivation, interference with bacterial membranes, and reduced adhesion-capabilities of bacteria to bladder epithelium or catheter material due to effects on biofilm formation and quorum sensing (7, 12 – 14). NTX^R^ *E. coli* generated by *in vitro* selection with low frequency, was shown to carry stable mutations in *emrR (mprA*), the transcriptional repressor of the EmrAB efflux pump, confirming EmrAB-TolC as reason for decreased NTX susceptibility (15). Additional mutations could be identified in *lon* and *marR*, suggesting also a role of AcrAB (16); however, in the same study knockout, mutants of *marR* and *acrRAB* showed no change in susceptibility (15). In light of an inevitable rise of resistance, it remains uncertain, why efflux-mediated resistance mechanisms caused by simple mutations of the negative regulator should not occur in the clinical setting, especially when these mutations are not associated with a significant fitness loss (15). It can be hypothesized that the moderate use of NTX, its efficient excretion into urine and currently not fully understood mode of action are a reason for low occurrence of NTX^R^ *in vivo*.

Originally, NTX was approved before the implementation of standardized regulations; hence, we sought to test NTX directly in comparison to other UTI reference drugs including NFT, Ciprofloxacin (CIP), FOS, PIV (as Mecillinam), and TMP as a head-to-head comparison in standard microbiological evaluation and to test its potential for resistance development. We generated resistant mutants by adaptation to reflect the *in vivo* situation and performed genomic, proteomic, and metabolic analyses of NTX^R^ mutants in combination with *in vivo* assessment in zebrafish larvae to better understand the consequences of NTX^R^ and its low occurrence in clinical settings.

## RESULTS

### Nitroxoline shows superior *in vitro* activity against a panel of common UTI pathogens

For a general overview on the antibiotic efficiency spectrum, we have tested NTX together with common UTI reference drugs NFT, FOS, PIV (as Mecillinam), CIP, and TMP against six different species (*E. coli*, *K. pneumoniae*, *Enterococcus faecalis*, *Proteus mirabillis*, *Pseudomonas aeruginosa*, and *Staphylococcus aureus*) combining a small panel of laboratory strains and freshly isolated MDR clinical isolates from uncomplicated UTI. The minimal inhibitory concentration (MIC) was determined using standard broth microdilution (NTX, NFT, CIP, and TMP) or agar dilution (FOS and PIV) (Table 1). NTX showed good activity against all species except *P. aeruginosa* and no loss of activity was seen for any of the clinical isolates when compared against the clinical breakpoint of 16 µg/mL for *E. coli* (17). For the ease of classification, we applied the breakpoint of *E. coli* also to the other species, for which there are none available due to limited clinical data. For all other reference drugs, resistance phenotypes (MIC above clinical breakpoints of susceptibility) against several tested strains could be observed. TMP and PIV were ineffective against almost all clinical isolates, while NFT was ineffective against both *P. mirabilis* and *P. aeruginosa*. For two isolates (*K. pneumoniae* MHH84299 and *P. mirabilis* MHH82530), NTX was the only drug that could have been considered for treatment.

**TABLE 1:** Antibiotic spectrum of common UTI drugs tested with a small panel of laboratory strains and clinical UTI isolates according to EUCAST guidelines ISO20776-1:2019^*d*^

MIC [µg/mL]	NTX	NFT	FOS^*a*^ ^,*b*^	PIV^*a*^	CIP	TMP
*E. coli* ATCC25922	4	8	1	0.125	0.008	1
*E. coli* MHH79495^*c*^	4	16	2	2	0.016	**>64**
*E.coli* MHH85227^*c*^	2	16	2	0.25	**64**	**>64**
*K. pneumoniae* DSM681	2–4	16–32	4	0.0625	0.004	0.5
*K. pneumoniae* MHH84274^*c*^	4	64	8	8	**1**	**>64**
*K. pneumoniae* MHH84299^*c*^	4	**>64**	**>64**	**>64**	**64**	**>64**
*E. faecalis* ATCC29212	16	8–16	**32**	**>64**	1	0.25
*E. faecalis* MHH83300^*c*^	8	8	**64**	**>64**	0.5	0.125
*P. mirabilis* DSM4479	16	**>64**	**64**	**16**	0.03125	2
*P. mirabilis* MHH82530^*c*^	16	**>64**	**32**	**>64**	**8**	**>64**
*P. aeruginosa* DSM27853	**32**	**>64**	4	**>64**	**0.125**	**>64**
*P. aeruginosa* MHH84389^*c*^	**64**	**>64**	**64**	**>64**	**8**	**>64**
*S. aureus* ATCC29213	2	16	2	**64**	**0.25**	2
*S. aureus* MHH80207^*c*^	4	32	2	**>64**	**>64**	1
*S. aureus* MHH85297^*c*^	8	16	**64**	**>64**	**>64**	0.5

^*a*^ Agar dilution method.

^*b*^ Supplemented with 25 mg/L glucose-6-phosphate.

^*c*^ Clinical isolate from Hannover Medical School (Germany), check SI for more details.

^*d*^ Bold numbers indicate non-susceptibility according to EUCAST (For NTX, *E. coli* breakpoints were used as approximation for all species).

### Nitroxoline resistance does not occur using standard procedures

We have used four well-characterized laboratory strains to determine their frequency of resistance (FoR) against different antibiotics at 4× or 8×MIC (Table 2). Bacterial strains were grown overnight and then diluted to adjust the respective inoculum. Single colonies were counted and 10 random clones were picked each to confirm a change in antibiotic susceptibility. With mutation frequencies of 10^−5^, TMP and PIV had very high mutation rates *in vitro* at 4× and 8×MIC. *E. coli* and *K. pneumoniae* both showed quite high FoR to FOS, but no mutants were acquired using *S. aureus*. For CIP, FoR ranged from 10^−7^ in *E. faecalis* to 10^−9^ in *K. pneumoniae* indicating medium-level FoR. For NFT, FoR was very low; mutants could only be generated in *K. pneumoniae* and *S. aureus*, while no clones could be isolated from the other two species (FoR < 10^−10^). In this setup, no resistant colonies could be generated using NTX, resulting in a FoR below 10^−10^ to 10^−11^ for all four strains at 4× or 8×MIC.

**TABLE 2:** Frequency of resistance of common UTI drugs against different laboratory strains

Frequency of resistance	NTX		NFT		FOS		PIV		CIP		TMP	
	4×	8×	4×	8×	4×	8×	4×	8×	4×	8×	4×	8×
*E. coli* ATCC25922	<1.0 × 10^−10^	<1.0 × 10^−10^	<8.1 × 10^−10^	<8.1 × 10^−10^	3.9 × 10^−7^	4.4 × 10^−8^	2.2 × 10^−5^	2.2 × 10^−5^	4.8 × 10^−8^	1.3 × 10^−8^	8.1 × 10^−5^	1.5 × 10^−5^
*K. pneum*. DSM681	<5.0 × 10^−11^	<5.0 × 10^−11^	1.2 × 10^−9^	6.1 × 10^−10^	3.5 × 10^−6^	1.3 × 10^−6^	nd^*a*^		3.1 x-10^−9^	2.3 × 10^−9^	>6.2 × 10^−5^	>6.2 × 10^−5^
*E. faecalis* ATCC29212	<8.6 × 10^−11^	<8.6 × 10^−11^	<8.6 × 10^−11^	<8.6 × 10^−11^	nd^*a*^		nd^*a*^		3.8 × 10^−7^	2.5 × 10^−7^	4.5 × 10^−6^	2.0 × 10^−6^
*S. aureus* ATCC29213	<3.1 × 10^−10^	<3.1 × 10^−10^	<3.1 × 10^−10^	3.9 × 10^−10^	<2.8×10^−10^	<2.8×10^−10^	nd^*a*^		7.7 × 10^−8^	<1.7 × 10^−9^	4.7 × 10^−6^	2.4 × 10^−6^

^*a*^ nd: not determined.

### Nitroxoline resistance can only be generated by slow adaptation

**Fig 1:**
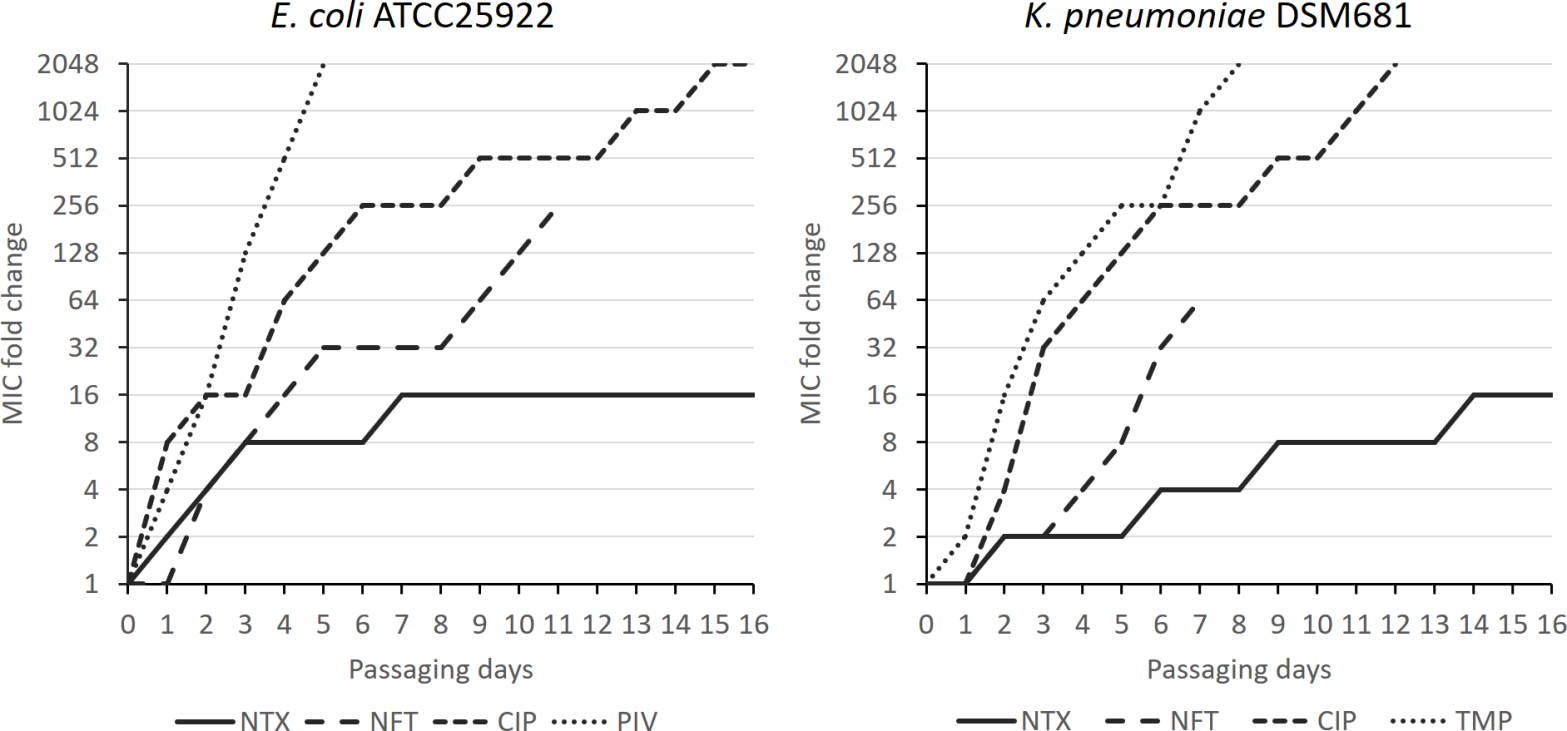
NTX resistance can only be slowly generated using adaptation. Strains were exposed to different concentrations of selected UTI drugs and passaged after 24 h incubation. The culture sample with the highest antibiotic concentration that still showed growth was used to inoculate the next sample. Antibiotic susceptibility is expressed as fold change in relation to standard MIC values as displayed in Table 1.

*In vivo*, antibiotics are usually administered multiple times, metabolized and excreted. Therefore, bacteria are exposed to varying concentrations and thus, changing selective pressure. Consequently, antibiotic concentrations might not be sufficient to prevent growth and bacteria can adapt to the respective compounds. We aimed to resemble bacterial growth at sub-inhibitory concentrations using exposure at sub-MIC with multiple rounds of sub-culturing. After long-term exposure and adaptation (LEA), we have been able to generate resistant mutants using *E. coli* ATCC25922 and *K. pneumoniae* DSM681 with NTX and three selected reference antibiotics (Fig. 1). FOS was not tested due to single dose regimen in human application. Reference values were standard MICs as listed in Table 1. Both PIV and TMP resulted in high-level resistance (MIC shift >1,000×) within only a couple of days, whereas it required approximately 2 weeks for both strains to reach a 1,024-fold shift in MIC against CIP (256-fold after 1 week). Within the first 3 days, NFT and NTX showed a similar resistance development before resulting in a 32-fold and 16-fold shift, respectively, in *E. coli* and a 64-fold and only 4-fold shift, respectively, in *K. pneumoniae* after 1 week. NFT had to be stopped at the indicated time points due to reaching its solubility limit. In *E. coli*, NTX resistance did not increase any further and remained at 16-fold even after 16 days of constant antibiotic pressure. NTX^R^ also plateaued in *K. pneumoniae* at 16-fold after 14 rounds of passaging. As expected based on *in vitro* FoR, resistance development against NFT and NTX was significantly slower compared to PIV, CIP, and TMP. Importantly, NTX resistance could not be pushed further than 16-fold in both *E. coli* and *K. pneumoniae*. All isolated NTX^R^ mutants are displayed in Tables 3 and 4, respectively. Additionally, NTX^R^ mutants were tested after serial passaging without selective pressure for 10 days, but no reversion of resistance could be observed (data not shown).

**TABLE 3:** Overview of NTX^R^ *E. coli* strains generated by adaptation^*a*^

MIC	NTX	
	[µg/mL]	Fold change
Ec25922_WT	4	1
Ec_M1^*b*^	64	16
Ec_M2	64	16
Ec_M3	64	16
Ec_M4	32	8
Ec_M5	32–64	8–16
Ec_M6	64	16
Ec_M7^*b*^	32	8
Ec_M8	64	16
Ec_M9	32	8
Ec_M10^*b*^	32–64	8–16
Ec_M11	32	8
Ec_M12	32	8
Ec_M13	32	8

^*a*^ MIC is displayed in µg/mL and normalized against *E. coli* ATCC25922 wildtype (fold change). Strains were grown in MHBII containing slowly increasing sub-inhibitory NTX concentration and isolated after 10 days of constant exposure (Ec_M1-13).

^*b*^ Used for follow-up eperiments.

**TABLE 4:** Overview of NTX^R^ *K. pneumoniae* strains generated by adaptation^*a*^

MIC	NTX	
	[µg/mL]	Fold change
KP681_WT	2	1
Kp_M1	32	16
Kp_M2	32	16
Kp_M3	16–32	8–16
Kp_M4	16	8
Kp_M5	32	16
Kp_M6	16	8
Kp_M7	16	8
Kp_M8	32	16
Kp_M9	32	16
Kp_M10	32	16
Kp_M11	32	16
Kp_M12	32	16
Kp_M13	16–32	8–16
Kp_M14	32	16
Kp_M15	32	16
Kp_M16	32	16
Kp_M17	32	16

^*a*^ MIC is displayed in µg/mL and normalized against *K. pneumoniae* DSM681 wildtype (fold change). Strains were grown in MHBII containing slowly increasing sub-inhibitory NTX concentration and isolated after 10 days of constant exposure (Kp_M1-17).

### Efflux-related genes are the major sites of mutation after prolonged NTX exposure

All isolated NTX^R^ LEA-mutants were subjected to whole-genome sequencing. Subsequent analysis again revealed the *emrRAB* efflux system as major site of mutation in *E. coli* (Table 5). Herein, we also confirmed this for NTX^R^ *K. pneumoniae* isolates (Table 6); hence, all NTX^R^ clones carry mutations in *emrR* with Ile27Lys being the most frequent site of mutation (9 out of 17 independent NTX^R^ *K. pneumoniae* clones). Additional mutations could be observed in pleiotropic regulators *marR* (*E. coli*) or *ramR* (*K. pneumoniae*). With the multidrug efflux pump OqxRAB showing mutations after prolonged exposure, a third efflux system could be identified in *K. pneumoniae*. Clones carrying *oqxR* mutations also showed cross-resistance to other reference antibiotics (Table S1). Further, we also identified the *lon2-*encoded protease bearing mutations in 10 out of 13 *E. coli* clones, whereas this mutation was not found in *K. pneumoniae*. So far undescribed for NTX is *envZ*; here, nine clones carried six different single-nucleotide polymorphisms (SNPs), whereas downstream effects and their role in resistance were so far unclear. With *iscR* being mutated, some *K. pneumoniae* mutants also carry mutations in genes encoding for proteins playing a role in iron metabolism and iron-sulfur cluster assembly.

**TABLE 5:** Mutation sites in NTX^R^ *E. coli* indicate an efflux-mediated mode of resistance^*a*^ ^,*b*^

	*mprA 2 (emrR)*	*emrA*	*emrB*	*marR*	*lon 2*	*envZ*
Ec_M1	Leu95Pro	−49C > T	Ile480Leu		Asn417Lys	Val54Glu
Ec_M2	Leu95Pro	−49C > T	Ile480Leu		Asn417Lys	Val54Glu
Ec_M3	Leu151Pro	Ala5Ala^*c*^			Tyr141∆	
Ec_M4	Glu55Stop				Gly359Arg	Ile86Ser Arg397Cys
Ec_M5		−11A > T			Ser101-Ala114	Val241Gly
Ec_M6	Leu151Pro Arg90Gly	Pro10Pro^*c*^			Leu175∆	Val54Glu Pro248Ala
Ec_M7	Glu55Stop			Asn89Thr		
Ec_M8	Ala92Asp				Glu636Stop	
Ec_M9	Glu55Stop				Asn417Lys	Ile86Ser
Ec_M10	Glu55Stop	Ala5Ala^*c*^		Ala70∆		Leu43Ile
Ec_M11	Glu55Stop				Asn417Lys	Ile86Ser
Ec_M12		Gln11Lys		Gly104Asp		
Ec_M13	Glu55Stop				Asn417Lys	Arg397Cys

^*a*^ All mutants show an 8–16× reduced susceptibility to NTX.

^*b*^ ∆: deletion; Leu95Pro: single-nucleotide polymorphism with amino acid exchange as indicated; -49C>T: exchange of C to T 49 bp upstream of coding region.

^*c*^ Silent mutation.

**TABLE 6:** Mutation sites in NTX^R^ *K. pneumoniae* indicate an efflux-mediated mode of resistance^*a*^ ^,*b*^

	*mprA (emrR)*	*emrA*	*ramR*	*bepF (oqxA)*	*oqxR*	*iscR*
Kp_M1	∆5(224)		Ala40Thr			Leu129Gln
Kp_M2	∆5(224)		Ala40Thr			Leu129Gln
Kp_M3	Trp100Gly		Thr119ins			
Kp_M4	Ile27Lys		Thr119Pro			
Kp_M5	Leu139Gln Arg176His		Leu44Arg			
Kp_M6	Leu139Gln		His169Ala			
Kp_M7	Leu64Gln				∆87(314)	
Kp_M8	Ile27Lys				Ins24(174)	
Kp_M9	Ile27Lys				Gln11Pro	
Kp_M10	Ile27Lys				Leu31Gln	Tyr41Cys
Kp_M11	Ile27Lys				Ins24(204)	
Kp_M12	Ile27Lys	∆9(13)	Glu164Lys			
Kp_M13	Gln51Stop		∆82(426)			
Kp_M14	Gln51Stop			−155G > T	∆30(36)	
Kp_M15	Ile27Lys					
Kp_M16	Ile27Lys		Complex			
Kp_M17	Ile27Lys			−155G > T	Ins24(176)	

^*a*^ All mutants show an 8–16× reduced susceptibility to NTX.

^*b*^ ∆: deletion; brackets: position on nucleotide level based on ORF; number in combination with brackets: how many nucleotides deleted or inserted (ins). Trp100Gly: single-nucleotide polymorphism with amino acid exchange as indicated. For Kp2_NTX_14, the *ramR* modifications combine several deletions or insertion and SNPs.

### NTX resistance significantly affects bacterial fitness and *in vivo* virulence

After mutant generation, we were interested in the potential fitness costs caused by the observed mutant genotypes and analyzed all generated NTX^R^ *E. coli* clones using regular growth curves as well as microcalorimetry (Symcel, CalScreener), an innovative label-free technique to directly monitor the metabolic response of bacterial cells. In growth curves, most resistant clones displayed similar growth between 4 and 8 h, but optical density at the end of the experiment as a measure of biomass was slightly lower in almost all cases compared to wild type (Fig. S1). Both Ec_M7 and Ec_M10 showed delayed growth indicated by a prolonged lag phase and a reduced growth rate (*P* = 0.0444 and <0.0001, respectively), but ultimately Ec_M7 reached a final density comparable to wild-type cells. Overall, the majority of strains showed a similar growth behavior compared to the wild type with growth rates being statistically indifferent for 11 out of 13 mutants (Fig. S1), although a trend to slightly slower growth rates was visible.

**Fig 2:**
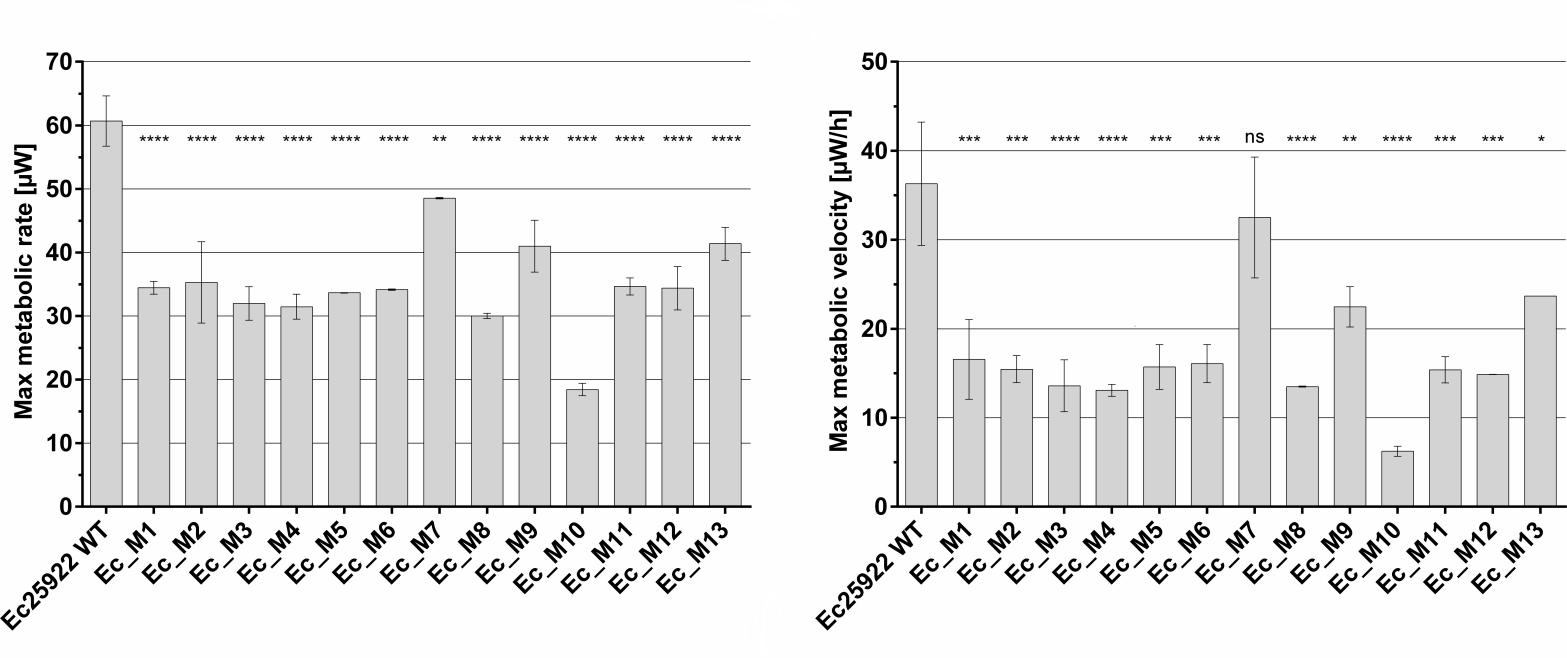
Microcalorimetry reveals significant impact of NTX^R^ on metabolic activity of *E. coli*. Peak heat flow (max. metabolic rate, left) and maximal change in heat flow (max. metabolic velocity, right) were determined following CalScreener analysis (*n* = 2), mean and standard deviation are shown. Samples were compared using one-way ANOVA with Dunnett’s multiple comparison test in GraphPad; *P < 0.05, **P < 0.01, ***P < 0.001, ****P < 0.0001.

In order to investigate these potential differences in more detail, we used microcalorimetry to assess the metabolic profile of NTX^R^ *E. coli* based on heat released during growth and now, striking differences could be observed compared to wild-type cells (Fig. S2). We quantified both maximal metabolic rate (peak heat flow, MMR) and maximal metabolic velocity (change in heat flow, MMV) using the CalScreener (Fig. 2). Both were reduced in all mutants with Ec_M10 showing the lowest activity, while Ec_M7, Ec_M9, and Ec_M13 resulted in activity closer to wild type, confirming the findings from the growth analyses. Overall, microcalorimetry clearly confirms a strong influence of the mutations on the metabolic activity of all NTX^R^ mutants, which could not be concluded after sole growth rate analysis.

**Fig 3:**
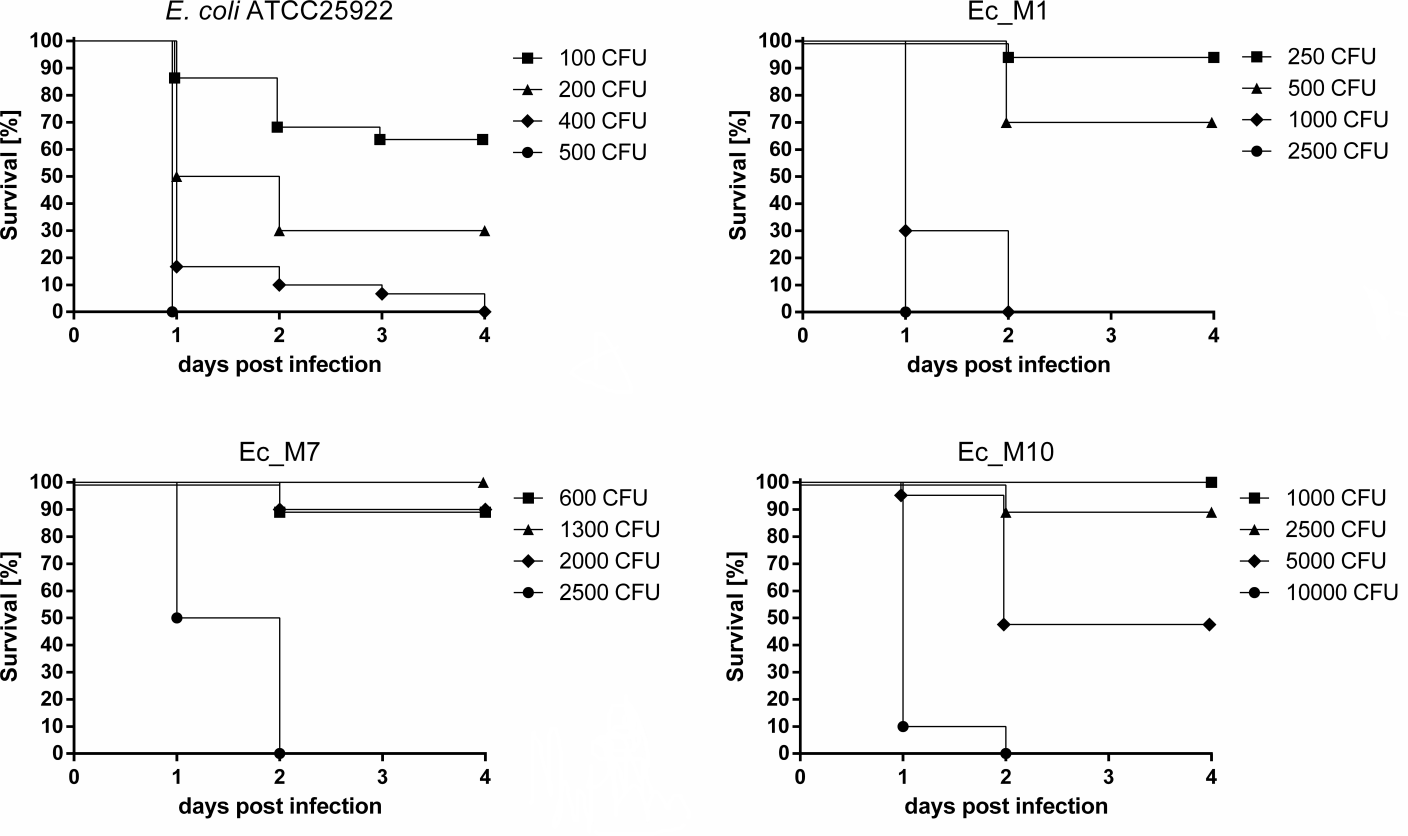
NTX^R^ diminishes ability of *E. coli* to cause an infection in zebrafish larvae. Zebrafish larvae were infected through the caudal vein 1 day post-fertilization (dpf), and survival was monitored until 4 days post-infection (dpi). Log phase cultures of respective strains were used to prepare infection solutions and inoculum was determined via injection into PBS followed by a viable cell count. Larvae injected with phenol red/PBS (1/1, vol/vol) served as controls (data not shown). Each line represents survival of 10–20 larvae per condition (WT 400 CFU, *n* = 30).

We then sought to check if this reduced metabolic capacity of NTX^R^ mutants also translates into reduced *in vivo* fitness and a potential loss of bacterial virulence in an infection model. Therefore, we used zebrafish larvae and systemically infected them 1 day post-fertilization (dpf) into the caudal vein using both *E. coli* wild-type and selected NTX^R^ mutants Ec_M1, Ec_M7, and Ec_M10. After infection, larval survival was noted at each day until 4 days post-infection (dpi) (Fig. 3). For the wild type, infection with 500 CFU resulted in 0% survival up to day 1 p.i. In contrast, infection with 500 CFU of Ec_M1, 600 CFU of Ec_M7, and 1,000 CFU of Ec_M10 resulted in 70%, 90%, and 100% survival, respectively. Ultimately, infection with CFU as high as 1,000 for Ec_M1, 2,500 for Ec_M7, and 10,000 for Ec_M10 were required to induce 0% survival. Survival gradually increased upon infection with lower doses of WT *E. coli* but only 70% survived up to 4 dpi upon infection with as few as 100 CFU. (Fig. 3). In contrast, infection with 2.5-, 6-, and 10-fold higher doses of Ec_M1, Ec_M7, and Ec_M10, respectively, still allowed survival of 90–100% of larvae. Thus, NTX^R^ diminishes the ability of *E. coli* to cause a severe infection *in vivo*.

### Nitroxoline resistance results in significant changes on proteome level

To gain a deeper understanding of NTX^R^ and potential physiological changes, we performed a full proteome analysis using *E. coli* ATCC29212 and mutants Ec_M1, Ec_M7, and Ec_M10. Bacteria were grown until early stationary phase (*n* = 4/strain), harvested, and prepared for full proteome extraction.

**Fig 4:**
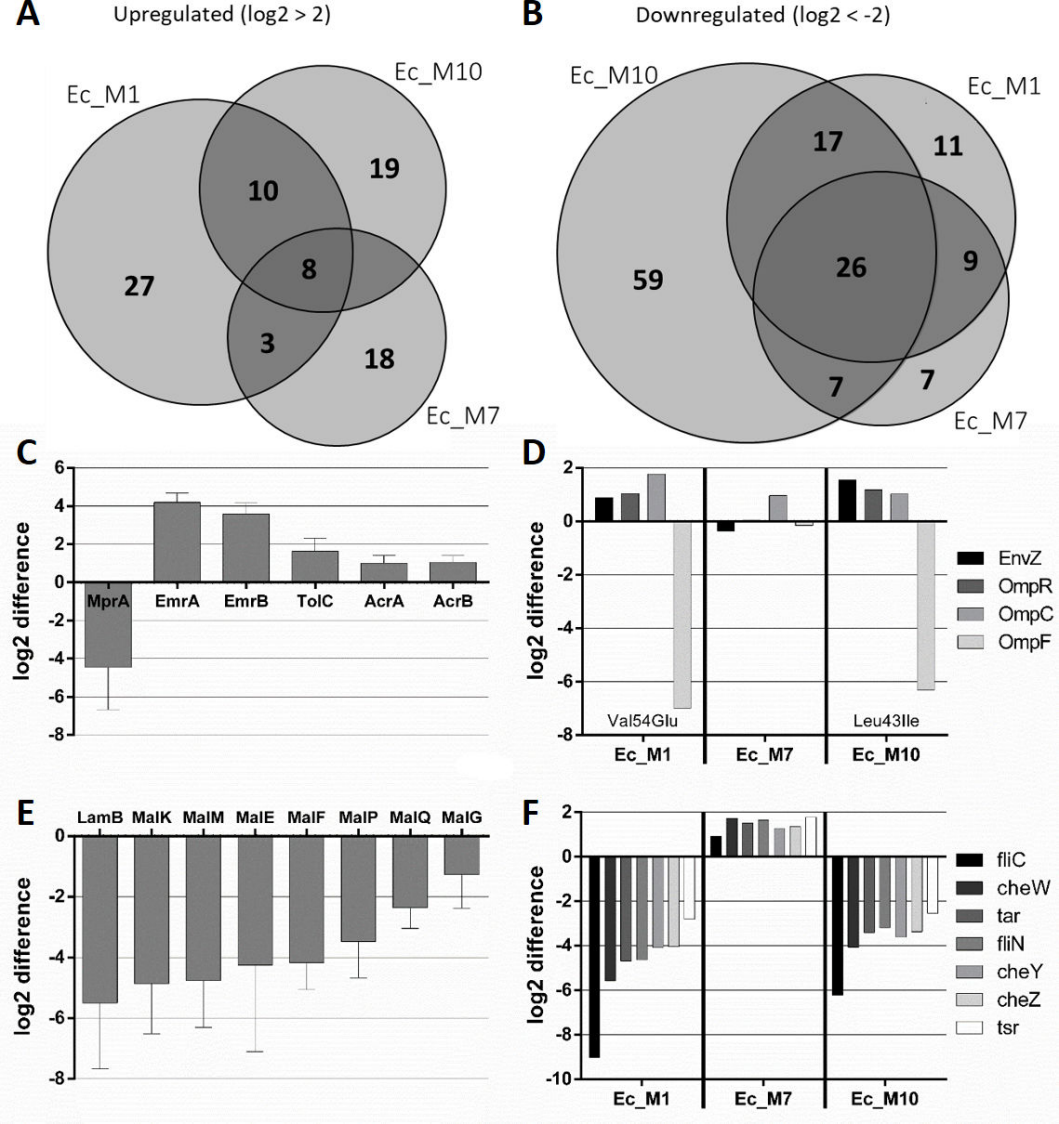
NTX^R^ strongly affects protein expression in *E. coli*. Venn diagrams of all three tested mutants and their significantly upregulated (A) or downregulated (B) proteins with the threshold indicated above the diagram. Expression differences in AMR-related efflux systems (C) EnvZ-OmpR system (D) maltose uptake system (E), and Chemotaxis-related proteins (F) are displayed as log2 difference from all three selected mutants compared to wild-type *E. coli* ATCC25922. For (C) and (E), mean and SD are shown. In (D), envZ mutations of the respective mutant are indicated within the diagram. Cells were harvested (*n* = 4 per condition) in early stationary phase and prepared for full proteome extraction followed by analysis via DIA-NN and Perseus.

After analysis, over 2,000 proteins could be identified in each sample providing excellent proteome coverage. Based on protein abundance, mutant samples were compared to the wild-type proteome to determine expression changes for each protein. The threshold was set to log2 >2 or <−2 (seen as fourfold up-/downregulated) and subsequently displayed as log2 difference. All significant expression changes can be seen in Fig. S3, individually displayed for each mutant. Forty-eight proteins were significantly higher abundant in Ec_M1, followed by 37 and 29 proteins in mutants Ec_M10 and Ec_M7, respectively, with some overlap between the individual mutants (Fig. 4A; Fig. S3) as determined using a Venn diagram (18). The only functional enrichment of upregulated proteins across all three mutants was related to efflux and combined EmrA (log2 = 4.205) and EmrB (log2 = 3.599), while differential expression of TolC, AcrA, and AcrB was below the chosen threshold (log2 = 1.639, 1.013, and 1.062, respectively) (Fig. 4C). MprA could not be detected in Ec_M7 and M10 at all (both carrying a Glu55Stop mutation); hence, we could confirm here that the introduction of the stop codon at Glu55 of *mprA* results in the halt of MprA production, whereas Leu95Pro only results in significantly reduced expression (Ec_M1). We showed here that the change in MprA expression most likely led to upregulation of the EmrAB efflux pump, whereas the AcrAB efflux system did not seem to play a major role in NTX^R^. Other proteins upregulated in all three mutants contain the nitroreductase NfsA (log2 = 3.13), the monooxygenase YbhW (3.83), or the zinc chaperone YeiR (2.90).

Regarding downregulated proteins, Ec_M10 showed the strongest overall response with more than 100 proteins affected, followed by Ec_M1 and Ec_M7 with 63 and 49 differentially expressed proteins, respectively, resulting in a total of 136 individual proteins showing fourfold lower abundance compared to wild type. While Ec_M1 and Ec_M7 shared over 80% of their differentially expressed proteins with at least one other mutant, Ec_M10 had more than 50% of downregulated proteins not found in the other two mutants indicating a larger proteomic difference (Fig. 4B). Overall, 20% (26/136) of proteins were significantly less expressed in all three mutants, while more than 40% (59/136) were reduced in at least two out of three mutants (Fig. S3). Of these, the String network indicated a significant enrichment of protein-protein interactions (*P* < 10^−16^) (Fig. S4).

Amongst other mutations, Ec_M1 and Ec_M10 carry a mutation in *envZ* (Val54Glu and Leu43Ile, respectively), which encodes a sensor histidine kinase as part of the two-component system EnvZ/OmpR controlling itself and porins OmpC/F. In these mutants, EnvZ was slightly upregulated, together with OmpR and the porin OmpC, while OmpF got drastically downregulated (Fig. 4D). As a follow-up, NTX permeability through these porins was predicted to be low (19) and both OmpF and OmpC single-knockout strains did not show any change in NTX susceptibility (Tables S2 and S3). Ec_M7 expressing wildtype *envZ*, did not have any change in expression of OmpF or OmpR, while a mild upregulation of OmpC was still detectable. A similar expression pattern across the three mutants could be seen for chemotaxis-related proteins. Both Ec_M1 and Ec_M10 showed significantly reduced expression in chemotaxis-related proteins like flagellin, while analysis of Ec_M7 did not indicate such downregulation at all (Fig. 4F). A motility assay confirmed reduced swarming behavior for flagella-deficient mutants M1 and M10 (Fig. S5).

Interestingly, all three mutants did not seem to express the maltose uptake system LamB-MalEFGK and other maltose-related proteins; hence, they were downregulated up to 150-fold with multiple proteins of this system being below the limit of detection in several mutant samples (Fig. 4E). Additionally, across two out of three mutants, a variety of other functional clusters could be identified among significantly downregulated proteins using String network and gene ontology (GO-term) analysis: mixed catabolic processes, amino acid metabolism, and several transport-related proteins (Fig. S4). Proteomics data clearly indicate metabolic reprogramming of NTX^R^ in *E. coli* and highlight the significant impact on bacterial metabolism despite allegedly simple mutations found in the genome.

## DISCUSSION

Nitroxoline is an antibiotic approved for the treatment of uncomplicated UTI caused by Enterobacteriales like *E. coli*, and it has been on the market for more than 50 years. To compare the general activity spectrum of the tested drugs, we conducted susceptibility testing using a limited selection of bacterial strains. Due to a lack of clinical data, breakpoints for NTX are only available for *E. coli* (17) and we applied these breakpoints as approximate measure to the other species and found good NTX activity against all species except *P. aeruginosa*. Of the other species, none would be characterized as resistant to NTX, although both *P. mirabilis* and *E. faecium* showed MICs close to the breakpoint. Notably, among our small panel, we identified two clinical isolates that exhibited susceptibility exclusively to NTX, while being resistant to all other tested drugs. Recently, Stolditis-Claus et al. demonstrated that NTX has the lowest resistance rate (3.9%, 48 out of 1,246 NTX data sets) among all commonly used antibiotics against Enterobacteriales (FOS 4.6%, NFT 11.7%, CIP 14.2%, TMP 24.2%, and PIV 30%) isolated from urine samples in Germany (total number of isolates *n* = 201,152) and concluded that its effectiveness has not decreased in recent years (9). Hof et al. (20) evaluated 477 isolates from urine samples and described excellent activity of NTX against *E. coli* with only 1 out of 277 isolates being resistant. They also found reduced activity against both *Pseudomonas* and *Enterococcus* but highlighted the use of NTX for 3MRGN or 4MRGN pathogens. Another study analyzed almost 700 *E. coli* samples (including 88 multidrug-resistant isolates) as well as 90 *P*. *mirabilis* isolates. Here, only three *E. coli* and four *Proteus* samples were found to be NTX^R^ (21). The same study also looked at multidrug-resistant *K. pneumoniae*; they identified 16 out of 50 isolates with an NTX^R^ phenotype indicating significantly higher resistance rates for *Klebsiella*. In light of AMR, NTX is experiencing a revival and was currently shown to have promising *in vitro* activity against other relevant pathogens such as *Acinetobacter baumannii*, *Neisseria gonorrhoea*, multidrug-resistant enterobacteriales, and *Candida* spp., suggesting a potential use for therapy of infections caused by these organisms (22 – 26).

The susceptibility of multidrug-resistant pathogens to NTX in combination with its relatively slow resistance development and good safety (27), makes NTX an ideal anti-infective drug for clinical application not only when no other options are available. We have shown here that *in vitro* resistance generation was challenging and occurred with a very low frequency, which is in line with existing literature (15). Only after adaptation, we were able to create low-level NTX^R^ mutants (8- to 16-fold shift in MIC compared to wild type) in both *E. coli* and *K. pneumoniae*. The level of resistance of these mutants could also not be increased any further even after prolonged exposure. Hence, it seems that only low-level resistance against NTX can be achieved with MIC values still below the maximal concentration of NTX determined in urine samples (28), making the reduced susceptibility of *in vitro* generated mutants less of a concern. Other common drugs used for the treatment of UTI were characterized by mutants developing at a higher frequency with high levels of resistance (over 2,000-fold shifts in MIC) which can explain increased resistance rates in clinical samples (9). Thus, in this head-to-head comparison, we found NTX clearly superior in terms of its resistance development properties *in vitro*. It is important to mention that *in vitro* FoR does not necessarily reflect *in vivo* resistance development, which might be especially prominent for Mecillinam, which is characterized by a very high FoR *in vitro* but still good efficiency in clinical application, where resistance development generally seems to be rare (29 – 31). However, the most recent study from Germany identified that almost 30% of tested UTI isolates were resistant to PIV (25,850 out of 86,371) (9) raising doubts regarding the clinical efficiency of PIV.

In previous studies, the multidrug resistance efflux system EmrAB-TolC has been identified as the main reason for NTX resistance in *E. coli,* and other mutations such as in *marR* (encoding for the master multiple antibiotic resistance regulator MarR) and *lon* (encoding an ATP-dependent protease) have also been described in the same study (15). The presence of either *marR* or *lon* mutations was associated with higher NTX^R^, whereas EmrAB only seemed to account for up to eightfold shifts in MIC of respective mutants. Interestingly, all low-level resistant (8–16× MIC shift) *E. coli* mutants that were generated here have either *marA* or *lon* mutations in combination with mutations in the *emrRAB* operon supporting this theory. However, we also found the nitroreductase NfsA significantly upregulated in all three mutants as well as monooxygenase YbhW. While NfsA is known to confer resistance to NFT (32), YbhW is mostly unknown and their role in NTX^R^ might be explored in the future. Interestingly, the zinc chaperone YeiR was also upregulated in all mutants, potentially hinting toward the indistinct ion-mediated mode of action of NTX, especially as previous data suggest that an increase in Zn^2+^ ions renders NTX ineffective against bacterial biofilm (14). We could confirm for the first time *emrR* (*mprA*) mutations also for all NTX^R^ *K. pneumoniae* mutants, however, mutations were found in neither *marR* nor *lon* despite of up to 16× resistance. Nevertheless, potential other secondary resistance mechanisms occurred in *K. pneumoniae*; with AcrAB (via RamR) and OqxAB, two additional mutated efflux systems were identified with the latter resulting in cross-resistance to other drugs such as TMP or CIP. While EmrAB in general is less commonly associated with resistance against antibiotics, OqxAB has been described to confer resistance to several antibiotics. This might explain the increased resistance rates of NTX in test panels of multidrug-resistant *Klebsiella* due to the shared efflux-related mode of resistance (33, 34).

In NTX^R^ *E. coli* mutants, the sensor histidine kinase EnvZ, necessary for cell homeostasis and iron transport, was found to carry several different mutations with so far undescribed consequences. EnvZ is part of a two-component regulatory system together with OmpR, involved in reciprocal regulation of membrane porins OmpC and OmpF depending on promoter interaction and potentially also connected to the expression of membrane-bound maltoporin LamB (35 – 37). Our full proteome analysis revealed a significant impact on these porins; hence, OmpC was slightly upregulated while OmpF and LamB were below the detection limit in mutant samples suggesting a significant downregulation of these porins. Usually, this constellation is described for situations of high cellular osmolarity and might be caused by NTX-induced ion chelation and subsequent intracellular accumulation (38). Other ion import proteins like FepA FecA, Fiu, YncE, and EntABF were also downregulated in NTX^R^ mutants, supporting this theory. However, no influence on other relevant proteins like FrdB, EfeO, Fur, or FeoAB was observed. Iron and its specific role in NTX antibacterial mechanism should be checked in the future. Recently, it was already demonstrated that genes related to iron acquisition are significantly upregulated in NTX-treated biofilm (14, 39). Interestingly, we identified *iscR*, a transcriptional regulator of iron-sulfur cluster biogenesis, as mutation site in *Klebsiella*, further underlining the potential connection between NTX and iron or iron-sulfur cluster proteins. The loss of outer membrane porins and the reduction of membrane permeability in general can also be associated with AMR and has been described as mode of resistance for several antibiotic classes (40 – 42). However, NTX permeability through OmpF or OmpC was predicted to be low and respective single-knockout strains did not show any change in NTX susceptibility (Tables S2 and S3). Importantly, mutant Ec_M7 expressing wild-type *envZ* did not have reduced abundance of OmpF but showed a mild upregulation of OmpC comparable to the other two mutants, suggesting another EnvZ-independent regulation of OmpC in our studies. For LamB, however, expression is only 8-fold reduced in Ec_M7 compared to 70- to 150-fold in mutants carrying *envZ* mutations, which further emphasizes the potential connection between LamB expression and EnvZ. This is the first time that EnvZ, OmpF, and LamB have been associated with NTX^R^, and further experiments are necessary to understand their role in NTX resistance. Our studies also suggest a potential link between EnvZ and bacterial chemotaxis since both mutants bearing SNPs in *envZ* can also be characterized by an alternated expression of flagellin and other chemotaxis-related proteins and also displayed reduced swarming behavior in our motility assay *in vitro*. Other studies have already hypothesized that OmpR might negatively regulate the expression of flagella (43 – 45) and have also connected NTX to changes in surface adhesion and pilus expression (14, 46), without giving an explanation. Our data allow the hypothesis that EnvZ is the key regulatory unit pathing the way for further research.

Previous studies have looked at NTX^R^ *E. coli* mutants by assessing their regular growth rate and found no impact for first-step mutants (up to 8-fold), while mild effects on fitness were described for second-step mutants (up to 16-fold) (15). We have applied a different methodology to study bacterial fitness and we show here that NTX^R^ is indeed associated with a significant fitness loss in all tested mutant strains, when analyzed via microcalorimetry, whereas standard growth rate analysis did only reveal a significant difference for 2 out of 13 *E. coli* mutants. Thus, the metabolic potential is affected without directly impairing bacterial growth, which also translates into reduced *in vivo* fitness as shown in a zebrafish larvae model. Here, we found reduced bacterial virulence—characterized by higher survival rates—following systemic injection of NTX^R^ *E. coli* mutants. Over 100 individual proteins were at least fourfold downregulated in NTX^R^ *E. coli* with the majority of proteins being connected to catabolic processes, metabolism, and molecular transport, indicating significant physiological changes, which were not directly obvious after whole-genome sequencing. At this stage, it is hard to pinpoint the exact mechanism behind the downregulation. It cannot be ruled out that mutations in the pleiotropic global regulators and two-component systems might play a role, but understanding of these regulatory mechanism are beyond the scope of this study. In addition to the general drop in metabolic activity, downregulation of flagella (FliCN and FlgH) and chemotaxis-related proteins (CheWYZ, Tsr, and Tar) could be a major reason for reduced virulence, as it was previously shown that these can be deemed essential during infection and are required for adhesion to the bladder epithelium (47 – 49). Xiao et al. already showed that mutations in *envZ/ompR* will render *Aeromonas hydrophila* less virulent resulting in higher survival rates of infected mice (45). This leads us to believe that our findings can also translate into reduced pathogenicity of NTX^R^ *E. coli* for rodents and humans. Importantly, the loss of flagella alone does not correlate with the fitness-loss observed in our larvae model underlining the complexity of the infection process.

Overall, we showed here, that NTX resistance, even when only low-level, indeed has drastic effects on bacterial metabolism and generally affects virulence and *in vivo* performance following systemic infection, which clearly offers an explanation for low occurrence rates of NTX resistance in clinical settings. The innovative framework we adopted allowed us to explore the subject from a fresh perspective yielding valuable insights and also showed that established techniques sometimes fail to detect important experimental outcomes.

Our study emphasizes the potential use of Nitroxoline in the context of AMR. We have shown that high-level resistance against NTX is unlikely as it could not be observed in our extended adaption experiment, and low-level resistance generally occurs only when sub-inhibitory concentrations are applied. Furthermore, such low-level resistant mutants are characterized by detrimental effects on bacterial fitness *in vitro* and *in vivo*. In case of NTX resistance, bacterial metabolism and virulence are impaired and cells will not be able to compete in an *in vivo* setup most likely preventing the spread and establishment of NTX resistance in humans.

## MATERIALS AND METHODS

### Strains and culture conditions

All laboratory strains used were purchased from the American Type Culture Collection (ATCC) or the German Collection of Microorganisms and Cell Cultures (DSMZ). Clinical strains were isolated from urine samples of hospitalized patients of Hannover Medical School (Germany). Bacterial routine culture was performed in cation-adjusted Müller-Hinton broth (MHBII) grown under ambient conditions at 37°C. All work was performed under biological safety level 2 (BSL2 laboratory) using standard microbiological techniques. Quality control was performed on regular basis using MALDI-TOF (Ultraflex III, Bruker).

### Minimal inhibitory concentration

MIC testing was determined using standard broth microdilution for NTX, NFT, CIP, and TMP or agar dilution for FOS and PIV according to EUCAST guidelines (ISO20776-1:2019). For all *in vitro* studies, Mecillinam was used rather than the oral pro-drug Pivmecillinam. For agar dilution, selective plates were inoculated using 10^4^ CFU/spot. For FOS, the agar was always supplemented with 25 mg/L glucose-6-phosphate. All antibiotics used were kept as single-use aliquots to avoid freeze-thaw cycles.

### Frequency of resistance

ON cultures were adjusted to 10^7^, 10^8^, 10^9^, 10^10^, and 10^11^ CFU/mL, respectively, prior inoculation of selective plates using 100 µL of culture suspension. After incubation, only plates with single colonies were counted and displayed as mean. At least 10 colonies were picked per antibiotic per strain to confirm resistance in a separate MIC assay. The exact inoculum was determined on non-selective agar and used for FoR calculation (resistant colonies/inoculum).

### Resistance by adaptation

LEA mutants were generated with *E. coli* ATCC25922 and *K. pneumoniae* DSM681 using a two-phase broth set up with slowly increasing the concentration of the respective antibiotic. In the first phase (Comparison Phase), five flasks were used per antibiotic per strain with concentrations ranging from 0.25× to 4×MIC. Flasks were always inoculated 1/200 from the flask containing the highest concentration with visible growth after 24 h incubation at 37°C (180 rpm) and concentration range was adjusted based on previous growth results. Procedure was repeated until high-level resistance, solubility limit, or plateau phase was reached. Once the general resistance development was determined, results from phase one were used and applied for Nitroxolin-only in phase 2 to generate at least ten independent mutants per strain that can be used for sequencing. Twenty individual culture flasks were kept per strain and exposed to same concentration, which slowly increased each day. During the entire procedure, a sample exposed to DMSO was used as control to account for random changes during the prolonged experiment and also used as sequencing control.

### Genomic analysis of NTX^R^ mutants

After resistance generation, genomic DNA was extracted using the QIAamp DNA Mini Kit (Qiagen) according to the manufacturer’s instructions in the QIAamp DNA Mini and Blood Mini Handbook, version 05/2016. Initial sample preparation was performed according to the recommended procedure for “Isolation of genomic DNA from a bacterial suspension culture” before switching to step 3 of the recommended protocol “DNA Purification from Tissues.” We optimised step 10 (additional centrifugation to dry column prior elution) and centrifuged for 30 min rather than only 1 min—we found this greatly improves the purity of the product after elution.

The gDNA of wild-type and mutant samples were sent for Illumina MiSeq (2 × 300 bp reads) and analyzed using Geneious Prime Version 2022. For short: paired reads were imported and mapped against the respective annotated reference genome retrieved from ATCC Genome Portal for *E. coli* ATCC25922 and *K. pneumoniae* ATCC10031 (DSM681). After mapping, the consensus sequences were generated and aligned against the annotated reference genome (Progressive Mauve Algorithm) with a match seed weight of 15 and a minimum LCB score of 30,000. SNP calling was performed manually on the bases of both the original wild-type sample and the sequencing control grown for the entirety of the adaptation process. For *Klebsiella,* Mauve alignment was not possible due to multiple copies of highly similar stretches of rRNA hence *de novo* assembly of the reads had to be performed for each genome followed by LASTZ alignment against the reference genome.

### Analysis of *in vitro* fitness costs

For growth rate analysis, strains were grown ON and split into fresh medium prior analysis in a regular plate reader (Tecan M200 PRO) with its Tecan i-control software version 2.0.10.0. Cells were grown with orbital shaking in a U-bottom plate in triplicates and OD_600_ was read every 30 min. Evaluation was performed using GraphPad Prism and its nonlinear fit for logistic growth to determine rate constant *k*.

Metabolic analysis (Microcalorimetry) was performed using the CalScreener (SymCel). Cells were prepared from an ON culture and inoculated to OD = 0.001. Heatflow was measured using the CalView2 software, followed by subsequent analysis via the SymCel Calorimetry web app (Version 2021-04). For evaluation, all standard parameters were quantified and maximal metabolic rate (peak heat flow, MMR) and maximal metabolic velocity (max change in heat flow, MMV) were used for comparison. Experiments were performed two times, mean with SD was expressed in relation to wild type. Statistical analysis was done as indicated.

### Larval infection model

Husbandry of the adult zebrafish was performed according to internal protocols in accordance with the German Animal Welfare Act (§11 Abs. 1 TierSchG). Zebrafish larvae of the wild-type AB line were used for the study and always kept at 28°C in 0.3× Danieau’s solution. All experiments were done within the first 120 h post-fertilization (5 dpf). At 1 dpf, larvae were dechorinated using 1 mg/mL pronase and placed in low tricain solution for anaesthesia during injection. Bacterial suspension was prepared fresh from a log phase culture resuspended in PBS with 4% (mass/vol) polyvinylpyrrolidone (PVP). To aid visibility during injection, the suspension was diluted 1/1 (vol/vol) using phenol red (in PBS). Glass microinjection needles were produced in-house using standard glass capillaries and a micropipette puller (P-1000, Sutter Instruments). Injection inoculum was calibrated using standard oil injection procedure and validated using viable cell count on agar (in triplicates). As injection control, bacterial-free PBS/PVP/Phenol red solution was injected—when more than one larvae died in the control group, experiment was rejected. In general, a 10% death rate of the larvae was considered natural. Larvae were considered dead once no heartbeat could be recorded.

### Full proteome analysis

Sample preparation was based on previously published methods (50) and for exact procedure compare SI. Briefly: bacterial strains were grown to the early stationary phase, collected and lysed using 0.4% SDS and sonication. Protein concentration was determined using BCA assay and adjusted to 50 µg per sample, followed by acetone precipitation, trypsin digest, and desalting. After drying, peptides were resuspended to 0.5 µg/µL in 0.1% FA/water and prepared for Nano-LCMS/MS analysis via timsTOF Pro (Bruker), followed by analysis via DIA-NN and Perseus.

## Data Availability

Genomic data were deposited in the NCBI SRA database and can be accessed using the BioProject ID PRJNA1031934. The mass spectrometry proteomics data have been deposited to the ProteomeXchange Consortium via the PRIDE partner repository with the data set identifier PXD046609.

## SUPPLEMENTAL MATERIAL

Supplemental figures and tables spectrum.03079-23-s0001.docx Tables S1 to S3, Fig. S1 to S5, and supplemental methods.

Supplemental data set spectrum.03079-23-s0002.xlsx Antibiograms and sampling information for UTI clinical isolates.

ASM does not own the copyrights to Supplemental Material that may be linked to, or accessed through, an article. The authors have granted ASM a non-exclusive, world-wide license to publish the Supplemental Material files. Please contact the corresponding author directly for reuse.
