# Supplementary material for: Nitroxoline resistance is associated with significant fitness loss and diminishes in vivo virulence of Escherichia coli
Source: Microbiol Spectr. 2023 Dec 8;12(1):e03079-23. doi: 10.1128/spectrum.03079-23 (PMC10782962; doi:10.1128/spectrum.03079-23)
Supplement: Supplemental figures and tables — Tables S1 to S3, Fig. S1 to S5, and supplemental methods. [file spectrum.03079-23-s0001.docx]

| **Shift** | **NTX** | **NFT** | **TMP** | **CIP** |
| --- | --- | --- | --- | --- |
| Kp_DSM681 | 1  (2) | 1  (16) | 1 (0.25) | 1 (0.005) |
| Kp_M1 | 16 | 2 | 0.5 | 2 |
| Kp_M2 | 16 | 2 | 0.5 | 2 |
| Kp_M3 | 16-8 | 2 | 2 | 4 |
| Kp_M4 | 8 | 2 | 4 | 2 |
| Kp_M5 | 16 | 1 | 2 | 2 |
| Kp_M6 | 8 | 2 | 4 | 2 |
| Kp_M7* | 8 | 4 | 4 | 4 |
| Kp_M8* | 16 | 8 | >32 | >16 |
| Kp_M9* | 16 | 4 | 2 | 4 |
| Kp_M10* | 16 | 8 | 8 | 8 |
| Kp_M11* | 16 | 8 | >32 | >16 |
| Kp_M12 | 16 | 2 | 2 | 2 |
| Kp_M13 | 16-8 | 1 | 1 | 2 |
| Kp_M14* | 16 | >8 | >32 | >16 |
| Kp_M15 | 16 | 1 | 2 | 2 |
| Kp_M16 | 16 | 2 | 2 | 4 |
| Kp_M17* | 16 | >8 | >32 | >16 |
| *mutants carrying different *oqxR* mutations | | | | |

**Nitroxoline resistance is associated with significant fitness loss and diminishes *in vivo* virulence of *Escherichia coli***

**Supplementary Information**

**Table SI 1: NTX^R^ K. pneumoniae shows cross-resistance to other UTI drugs especially when they carry mutations in oqxR.** MIC values are displayed as shift normalized to wildtype value. The wildtype MIC is displayed in brackets in µg/mL.


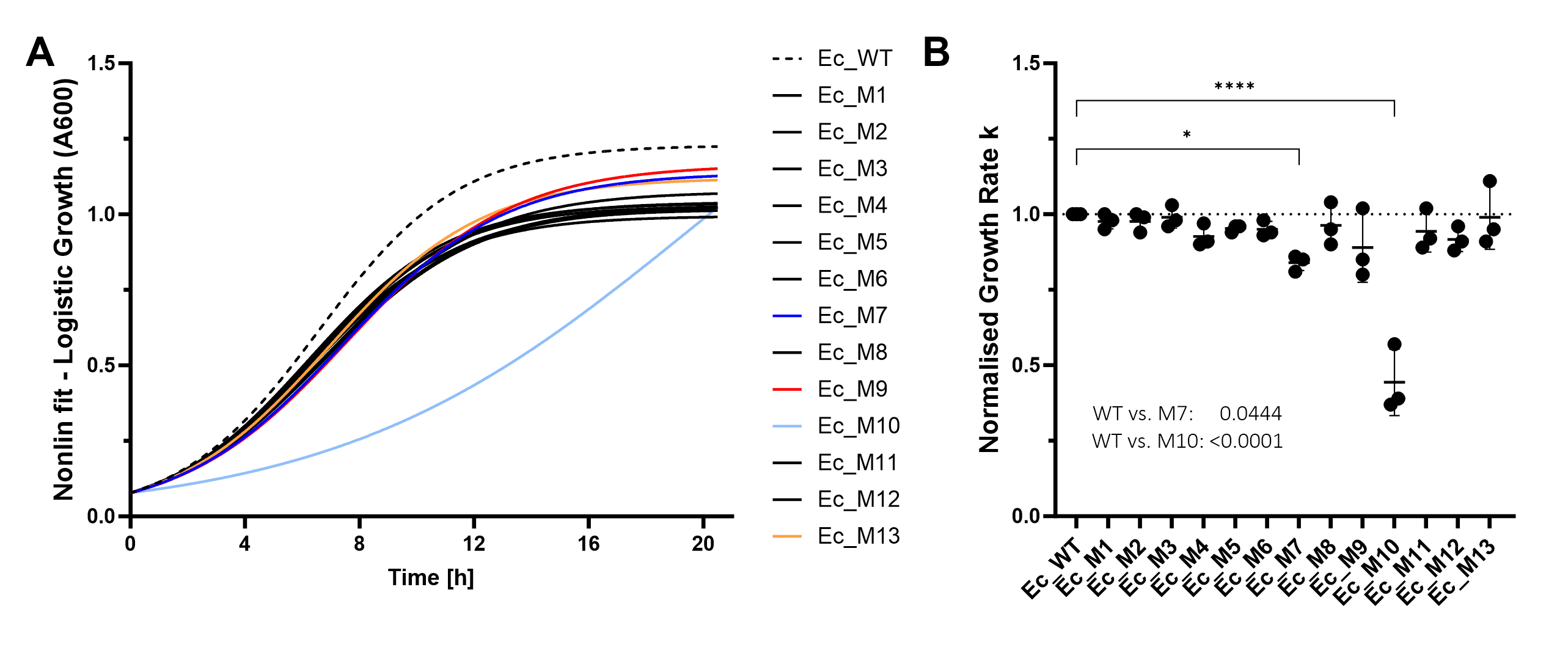


**Figure SI 1: Growth behavior of NTX^R^ E. coli.** Nonlinear fit of regular growth curves (A) with corresponding growth rates k (B). Experiment was performed in biological triplicates with two technical repeats each. To aid visibility, logistic growth is displayed as mean only across all experiments, with strains that appear different shown in colors, while all other mutants (similar to each other) were kept in black. In B, deducted growth rates are displayed as mean with SD, normalized to WT cells. Data was compared using an ordinary one-way ANOVA with multiple comparison test in GraphPad Prism.





**Figure SI 2: Microcalorimetric analysis (CalScreener) of NTX^R^ E. coli reveals striking difference to wildtype metabolism.** The last panel shows an explanation graph for the parameters maximal metabolic rate (as peak heat flow, MMR) and max. metabolic velocity (change in heat flow, MMV).





**Figure SI 3: Overview of full proteome analysis of NTX^R^ E. coli compared to wildtype E. coli ATCC25922.** Symbols indicate the three different mutants Ec_M1 (circle), Ec_M7 (square), and Ec_M10 (triangle). Proteins were sorted based on their clustering/functional enrichment in String Network and GO term analyses. Average and standard deviation are shown.


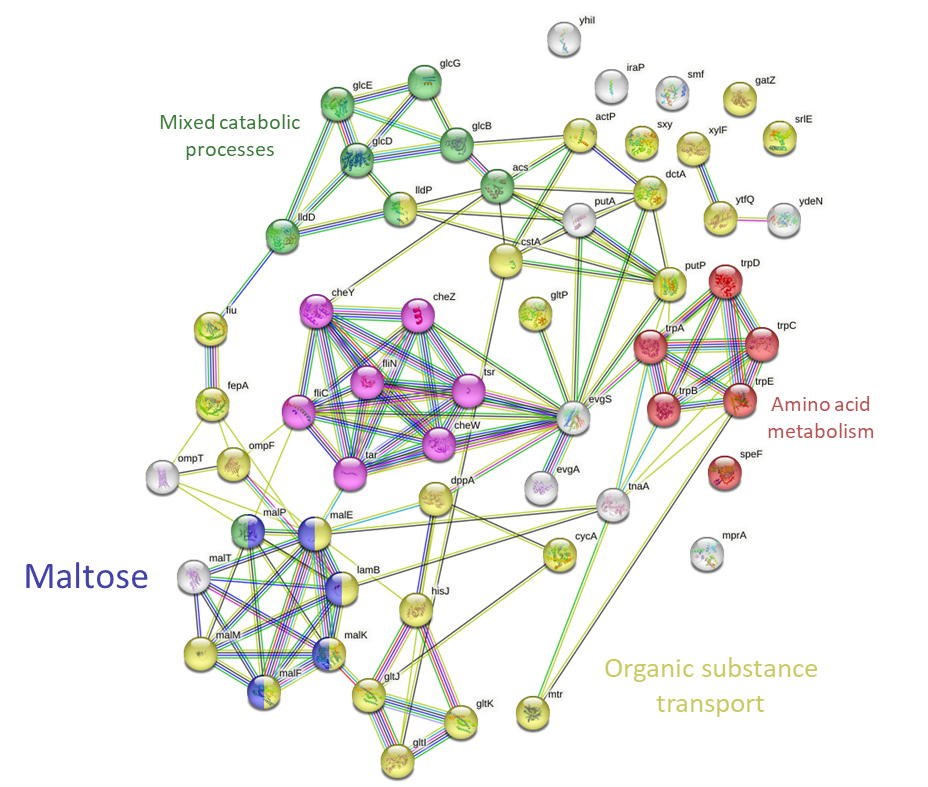


**Figure SI 4: String Network of Proteins downregulated in at least 2 out of 3 mutants.** Colors indicate different enriched clusters predicted by String Network and GO term analysis.

| **Pore** | **Steric term** | **Dipole moment** | **SF1 permeability [%]*** |
| --- | --- | --- | --- |
| **OmpF** | 4.97 | 0.27 | **24.24** |
| **OmpC** | 2.46 | 0.17 | **24.72** |
| *scoring function permeability with respect to glycine; Permeability <30% is considered poor^1^. | | | |

**Table SI 2: Low predicted permeability of NTX for major porins OmpF and OmpC**

**Table SI 3: E. coli knockout strains (KEIO collection) did not show any changes in NTX susceptibility**

| **Strain designation** | **NTX [µg/mL]** |
| --- | --- |
| *E. coli* BW25113 WT | 4 |
| *E. coli* JW3367 ∆*envZ* | 4 |
| *E. coli* JW2203 ∆*ompC* | 4 |
| *E. coli* JW0912 ∆*ompF* | 4 |
| *E. coli* JW3368 ∆*ompR* | 4 |
| *E. coli* JW3996 ∆*lamB* | 4 |


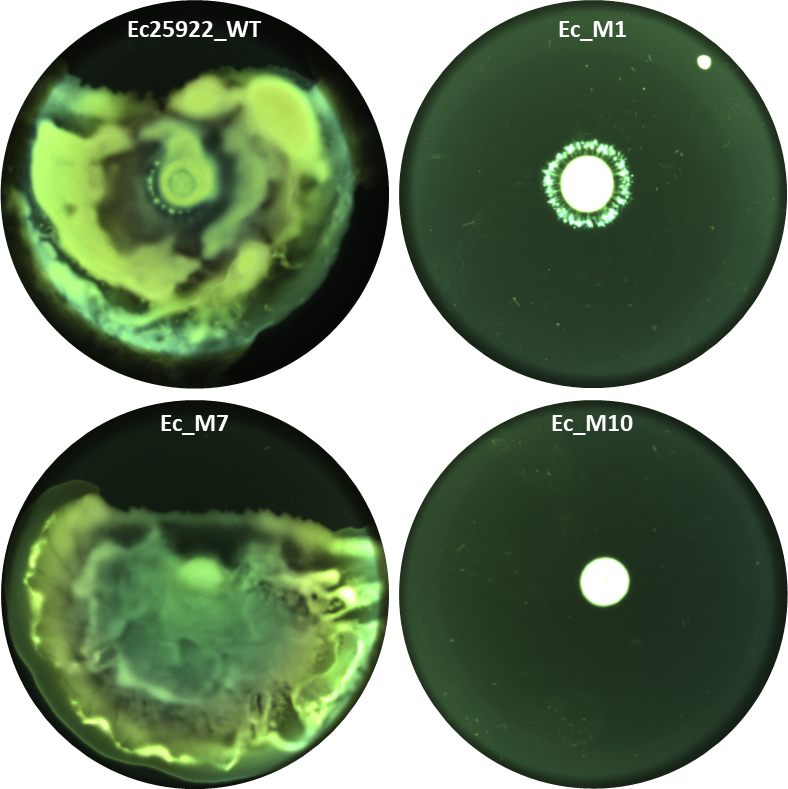


**Figure SI 5: Motility assay shows reduced swarming behavior of flagella-deficient mutants Ec_M1 and Ec_M10.** 2.5x10^6^ CFU were spotted on soft-agar plate (30g/L tryptic soy broth, 0.34% agar, 9 g/L glucose) and incubated for 24h at 37°C.

**Materials and Methods SI**

Full Proteome Analysis

Sample Preparation

An ONC was inoculated 1:100 in 4x5 mL fresh media and incubated at 37°C, 200 rpm, until the early stationary phase was reached (OD_600_ ~ 2). The samples were washed with 1000 µL cold PBS (6000xg, 5 min, 4°C) and the pallet was stored at -80°C until lysis. Lysis was done by adding 200 µL 0.4% SDS in PBS and three times sonication for 30 s at 50% intensity (Bandelin Sonoplus). Samples were centrifuged afterwards (20 min, 16900xg, RT). BCA assay was done according to manufactures instructions (Thermo Fisher) and proteome amount was adjusted to 50 µg per sample. Afterwards, the proteome was precipitated by adding 1 mL ice-cold acetone followed by incubation overnight at -20°C. Next, the samples were centrifuged (16900xg, 15 min, 4°C) and supernatant was discarded. Samples were washed two times by adding 1 mL ice-cold methanol, sonication for 10 s with 10% intensity and centrifugation (16900xg, 15 min, 4°C). Pellets were stored at -80°C.

Afterwards, samples were suspended in 200 µL X-buffer (7 M urea, 2 M thiourea, 20 mM HEPES, pH = 7.5) and 0.8 µL DTT (250 mM) per sample was added and incubated for 45 min, 25°C, 500 rpm (shaking). Next, 2 µL IAA (550 mM) were added and the mixture was incubated 45 min, 25°C, 450 rpm in the dark. Afterwards, additional 3.2 µL DTT (250 mM) were added and incubated for 30 min, 25°C, 450 rpm. 600 µL TEAB buffer (pH = 8) and 1 µL trypsin (0.5 µg/µL in 50 mM acetic acid) (MS grade, Promega) was added for digest overnight (>16 h) at 37°C, 450 rpm.

Digestion was stopped by adding 8 µL formic acid, vortex and centrifugation (13000 rpm, 3 min, RT). Samples were desalted on SepPak C18 columns 50 mg (Waters). First, columns were equilibrated by adding 1 mL ACN, 0.5 mL elution buffer (80% ACN, 0.5% FA) and 3x1 mL 0.1% TFA. Afterwards samples were loaded and washed 3x with 1 mL TFA, 250 µL 0.5% FA and eluted into 2 mL LoBind (Eppendorf) by adding 3x 250 µL elution buffer under vaccum. Samples were dried in the speedVac (45°C, V-AQ) before dissolving in 30 µL 1% FA by 10 min sonication in a water bath. Samples were filtered using 0.22 µm centrifugal filter (Merck Millipore) and prepared for LC-MS/MS analysis.

LC-MS/MS

Sample analysis was done by using nanoElute nano flow liquid chromatography system (Bruker, Germany) coupled with a timsTOF Pro (Bruker, Germany). Samples were loaded to the trap column (Thermo Trap Cartrige 5 mm) and washed with 6 µL 0.1% FA with a flow rate of 10 µL/min. Peptides were then transferred to the analytical column (Aurora Ultimate CSI 25 cm, IonOpticks) and separated by an gradient elution (0% to 3% in 1 min, 3% to 17% in 57 min, 17% to 25% in 21 min, 25% to 34% in 13 min, 34% to 85% in 1 min, 85% kept for 8 min) with H_2_O+0.1% FA (eluent A) and ACN+0.1% FA (eluent B) at a flow rate of 400 nL/min.

Captive Spray nanoESI source (Bruker, Germany) was used to ionize the peptides at 1.5 kV with 180°C dry temperature at 3 L/min gas flow. timsTOF Pro (Bruker, Germany) was operated in default dia-PASEF long gradient mode with TIMS set to 1/K0 start at 0.6 Vs/cm^2^, end at 1.6 Vs/cm^2^ with a ramp and accumulation time of 100 ms each and a ramp rate of 9.43 Hz. Mass range was set from 100.0 Da to 1700 Da with positive ion polarity. Dia-PASEF mass range was set to 400.0 Da to 1201.0 Da with a mobility range of 0.60 1/K0 to 1.43 1/K0 and a cycle time of 1.80 s. Collision energy for 0.60 1/K0 was set to 20.00 eV and for 1.6 1/K0 to 59.00 eV. Tuning MIX ES-TOF was used for calibration of m/z and mobility.

Data Processing

Data were processed using DIA-NN (version 1.8.1) and proteins were identified against Uniprot *E. coli* reference proteome (Proteome ID: UP000000625, downloaded 18/01/2023). Settings were used as default except precursor charge range was from 2 to 4. Cys-carbaidamethylation was set as fixed modification. “--relaxed-prot-inf” was added in additional options to allow further data processing with Perseus Software.

In Perseus (version 2.0.5.0) the values were transformed to their log2 and the replicates were grouped and filtered by three valid values in one group. Missing values were imputated by default settings and the differential protein abundance between different conditions were evaluated using students t-test. Cut-off for –log p-value was set to 1.3 (p-value = 0.05) and t-test difference 2 and -2. Proteins fitting these thresholds were significantly over or underexpressed compared to the wild type.

**Bibliography SI**

1. Acosta-Gutiérrez S, Ferrara L, Pathania M, et al. Getting Drugs into Gram-Negative Bacteria: Rational Rules for Permeation through General Porins. *ACS Infect Dis*. 2018;4(10):1487-1498. doi:10.1021/ACSINFECDIS.8B00108/ASSET/IMAGES/MEDIUM/ID-2018-00108T_M010.GIF
